# Disruption of the Human Gut Microbiota following Norovirus Infection

**DOI:** 10.1371/journal.pone.0048224

**Published:** 2012-10-30

**Authors:** Adam M. Nelson, Seth T. Walk, Stefan Taube, Mami Taniuchi, Eric R. Houpt, Christiane E. Wobus, Vincent B. Young

**Affiliations:** 1 Department of Internal Medicine, Division of Pulmonary and Critical Care Medicine, University of Michigan Medical School, Ann Arbor, Michigan, United States of America; 2 Department of Internal Medicine, Division of Infectious Diseases, University of Michigan Medical School, Ann Arbor, Michigan, United States of America; 3 Department of Microbiology and Immunology, University of Michigan Medical School, Ann Arbor, Michigan, United States of America; 4 Division of Infectious Diseases and International Health, Department of Medicine, University of Virginia, Charlottesville, Virginia, United States of America; Instutite of Agrochemistry and Food Technology, Spain

## Abstract

The gut microbiota, the collection of all bacterial members in the intestinal tract, plays a key role in health. Disruption of the indigenous microbiota by a variety of stressors, including antibiotic therapy and intestinal infections, is associated with multiple health problems. We sought to determine if infection with Norovirus disrupts the gut microbiota. Barcoded pyrosequencing of the 16S rRNA-encoding gene was used to characterize the stool microbiota in Norovirus-infected human patients (n = 38). While the microbiota in most infected patients (n = 31) resembled that seen in uninfected healthy controls, a minority of patients (n = 7) possessed a significantly altered microbiota characterized by reduced relative numbers of Bacteriodetes and a corresponding increase in Proteobacteria. In these patients, the increase in Proteobacteria was due to a single operational taxonomic unit (OTU) of *Escherichia coli*. We cultured *E. coli* from Norovirus-infected patients and characterized them using PCR-ribotyping and virulence factor analysis. Multiple ribotypes were encountered, but none possessed typical virulence factors commonly carried by enteropathogenic *E. coli* strains. Microbiota disruption and elevated Proteobacteria were not significantly correlated to patient age, gender, sampling time following illness onset, or overall gut inflammation. These results demonstrate that some patients have a disrupted microbiota following Norovirus infection, and therefore may be at elevated risk for long-term health complications.

## Introduction

The human gut microbiota is a complex bacterial community that is relatively stable over time [1], [2], [3]. Disruption of the microbiota can increase the risk for several health complications, including loss of colonization resistance against bacterial pathogens [4] and predisposition to autoimmune and allergic diseases [5]. Altered gut microbiota has also been linked to serious gastrointestinal disorders, including irritable bowel syndrome (IBS) [6], [7], [8]. A specific form of IBS, post-infectious IBS (PI-IBS) can develop when the microbiota is disrupted following gastrointestinal infection [9]. Disrupted communities, including those of patients with IBS, are often associated with highly elevated levels of Proteobacteria species, particularly *Escherichia coli* [10].

Norovirus (NoV) is a collective term for many related and highly contagious RNA viruses that can cause acute gastroenteritis in humans [11]. Each year in the United States, NoV infects an estimated 23 million individuals in all age groups [12], and is the leading cause of foodborne illness, with an estimated 5.5 million cases per year [13]. NoV is also a burden on health care facilities, accounting for 26% of all hospitalizations and 11% of all deaths directly attributable to foodborne illness [13]. Symptoms of NoV infection can be severe, with rapid onset diarrhea, abdominal pain, and vomiting, but illness duration is brief, typically lasting 12–60 hours [14]. However, asymptomatic infections or infections with atypical symptoms can also occur [15]. Human NoV is currently classified into three genogroups, with genogroup II, genotype 4 (GII.4) being the genotype most commonly linked to human disease [16].

While NoV infection is generally thought of as a self-limiting infection with no long-term sequelae in most individuals, there is recent evidence that infection can be associated with an increased risk of long-term health problems. One potential complication from NoV infection can be PI-IBS [17], [18]. While PI-IBS has been described following pathogenic bacterial infection [19], [20], the risk factors for developing PI-IBS following viral infection are not fully understood. Gut microbiota disruption may be one risk factor; however the relationship of viral gastroenteritis to disruption has not been extensively studied. Therefore we investigated if NoV infection, the most frequent cause of viral gastroenteritis [13], could also disrupt the gut microbiota.

This study used barcoded 454-pyrosequencing of the 16S rRNA gene to determine the microbiota diversity of human patients with NoV infection. Several host variables, including gut inflammation, age, and gender, were measured to determine if any were significantly associated with microbiota disruption. In addition, *E. coli* diversity in NoV-infected patients was investigated, using cultured *E. coli* isolates that were typed to determine variation both within and between patients, and a virulence gene assay to determine pathogenic potential. Our results demonstrated that some NoV-infected patients exhibit an altered intestinal microbiota, which may put them at elevated risk for health problems.

## Results

### Human Norovirus Patients and Healthy Control Subjects

NoV infected individuals (n = 38) included in this study were adults, sampled within a three month time span in Virginia, that were investigated as being involved in potential NoV outbreaks. A single sample was collected from each patient at one time point following illness, usually within two days of the onset of symptoms. Additional patient samples were unavailable for time points both before and after infection, and pre-existing health data for these patients was limited. Samples from multiple individuals within a given epidemiologic cluster were characterized. Genotyping of NoV stool samples verified all patients were infected with viral strains of genotype GII.4, but three patients were also co-infected with a Genogroup I strain (Table S1). As a comparator for microbiome analysis, 16S rRNA-encoding gene sequence data was obtained from a group of 22 healthy individuals that were part of the Human Microbiome Project (HMP) [21]. A summary of these patient groups (Table 1) includes comparisons of patient age, gender, and sampling time following illness onset.

**Table 1:** Comparison of Norovirus and healthy control patient pooled patient data.

	Norovirus Patients	Healthy Controls (HMP)
Num Samples(all genders)	38	22
Number of Males	18	10
Number of Females	19	12
Num of Unrecorded Gender	1	0
Average Age (years)	71.9	27.4
Median Age (years)	80	27
Age Range (years)	19–96	22–37
Avg. Age Male (years)	67.3	27.2
Avg. Age Female (years)	75.9	27.7
Average DPO (days)	1.40	n/a
Median DPO (days)	1	n/a
DPO Range (days)	0–9	n/a

Abbreviations: HMP, Human Microbiome Project (healthy control population); DPO, days post-onset (the time between symptom onset and sample collection); n/a, not applicable.

### Alteration of the Gut Microbiota in a Subset of Norovirus Patients

We obtained 16S rRNA-encoding gene sequence data from DNA isolated from the fecal samples of NoV-infected patients by barcoded 454 pyrosequencing. After quality-control processing, a total of 226,228 16S rRNA sequence reads were obtained from these NoV-infected patient samples. An additional 153,214 sequence reads were from 22 healthy HMP subjects. A summary of alpha diversity measurements for these samples is shown in Table S2. No significant differences were seen between the NoV-infected and HMP groups in terms of estimated sample coverage (p = 0.4031), or the number of 16S reads per sample (p = 0.1689), as measured by unpaired, two-tailed t-tests. Viral load, as determined by NoV genome copies per mL, did not significantly correlate to several patient variables, as measured by linear regression. These included lactoferrin level (p = 0.6805), patient age (p = 0.6001), and sampling time following illness onset (p = 0.8584). No significant correlation of viral load to Proteobacteria (p = 0.7494), Bacteriodetes (p = 0.6089) or Firmicutes (p = 0.8648) levels was found. Nor were patient gender and viral load (p = 0.2907) correlated, as determined by a two-tailed t-test. This confirms that the level of NoV detected in patient stool did not influence the level of gut inflammation, or the relative abundance of the most frequent bacterial phyla.

**Figure 1:**
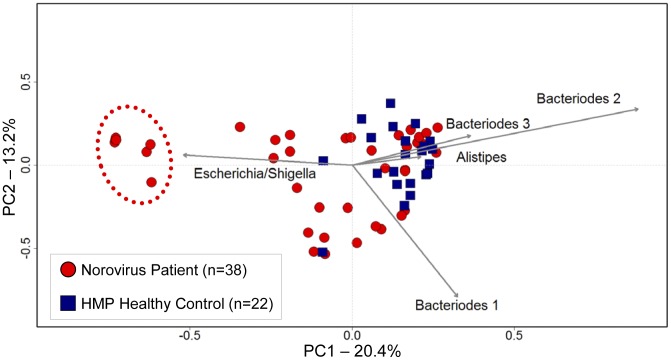
Some Norovirus patients have a highly divergent microbiota compared to controls. Principle coordinates analysis showed the community structure relationship between NoV and HMP patients. This ordination was generated using a Yue and Clayton-based distance matrix representing the relative abundance of OTUs in each community at a 3% OTU definition level. The community of each patient is indicated by a symbol. Arrows depict how the top five most frequently detected OTUs influence the location where each patient is represented. A subset of NoV-infected individuals display a highly divergent community characterized by decreased Bacteriodetes and an increased Proteobacteria. The patients circled represent the disrupted group, which are dominated by Proteobacteria. The majority of the elevated Proteobacteria in these patients was from a single OTU of *E. coli*.

To compare the community structure of the fecal microbiota of NoV-infected patients and HMP controls, principle coordinates analysis (PCOA) was used, based upon operational taxonomic unit (OTU) diversity (Figure 1). These data revealed that the community structure was not uniform, and a subset of NoV patients (n = 7) possessed a microbiota characterized by decreased Bacteriodetes and elevated Proteobacteria. Specifically, clustering was driven by a large increase of a single OTU, classified to the genus level as *Escherichia/Shigella*. In these patients, up to 99% of all 16S rRNA-encoding gene sequences were classified as Proteobacteria. We will refer to these seven patients as the disrupted group (DG), while the other 31 NoV patients that possessed a fecal microbiota similar to that in healthy controls will be called the undisrupted group (UG).

**Figure 2:**
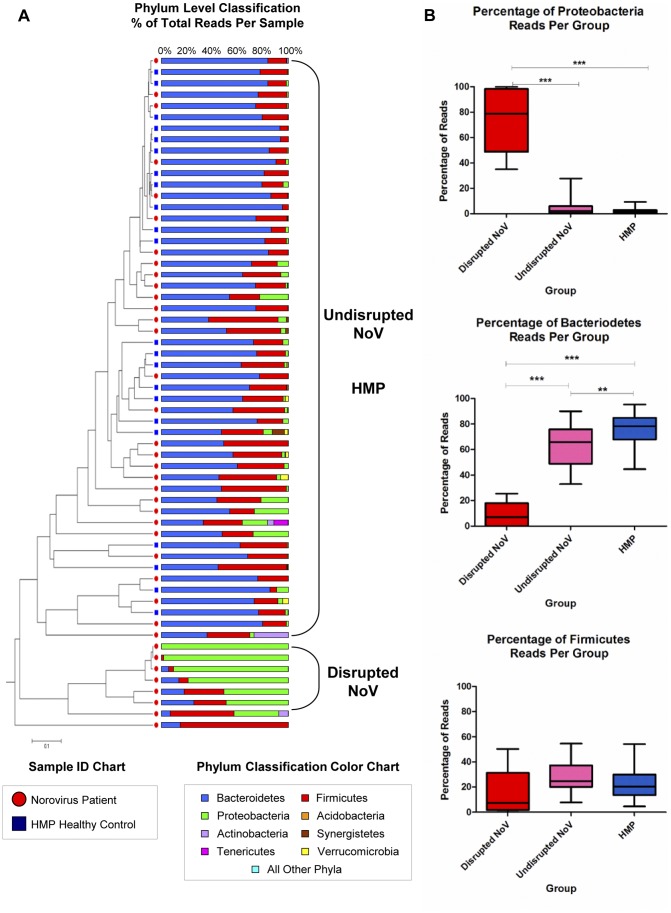
Patients infected with Norovirus show differential disruption. A) Dendrogram showing the phylogenetic relationship between samples, as calculated by the Yue and Clayton dissimilarity index at a 3% OTU level was paired with a phylum-level classification of the communities for each individual. B) Comparisons between different groups by a one-way ANOVA test showed that the disrupted patients have significantly less Bacteroidetes and significantly more Proteobacteria compared to the undisrupted and HMP patients. Abbreviations: HMP = Healthy control patient from the Human Microbiome Project.

Phylotype analysis further confirmed that the gut communities in DG patients were distinct from UG patients (Figure 2A). The community in DG patients had proportionately fewer Bacteroidetes (p = <0.0001) and significantly more Proteobacteria (p = <0.0001) compared to UG and HMP controls (Figure 2B), while Firmicutes levels were not significantly different between groups. A single NoV-infected patient showing high levels of Firmicutes and low levels of both Bacteroidetes and Proteobacteria was treated as an outlier and not included in either the disrupted or undisrupted groups.

**Figure 3:**
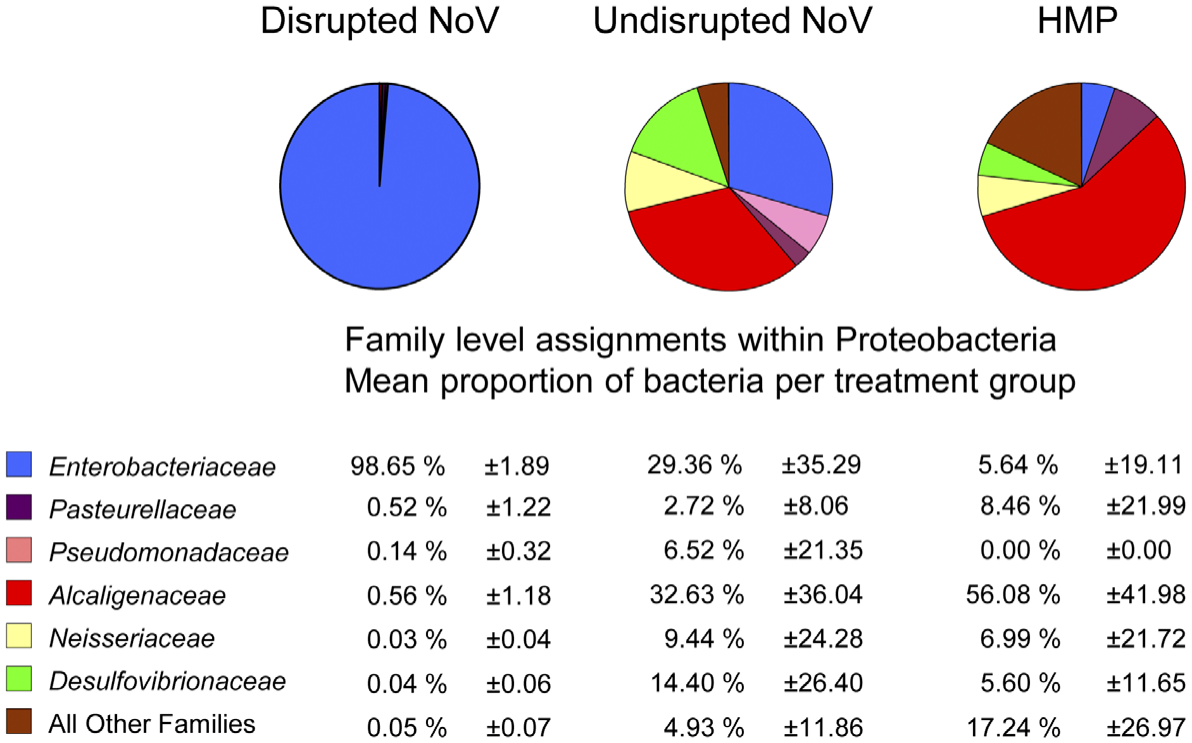
Patients with high Proteobacteria are dominated by high levels of *Enterobacteriaceae*. The average family level diversity within all Proteobacteria reads showed that the vast majority of Proteobacteria in disrupted patients was *Enterobacteriaceae*. Undisrupted and HMP patients had lower overall reads, and showed a higher diversity of reads within Proteobacteria, but lacked a single dominant bacterial family.

To determine if differences between groups were also present at the taxonomic level of bacterial family, sequence data from the Proteobacteria were specifically analyzed. These data revealed that DG patients had little diversity within the Proteobacteria, with the vast majority of reads classified as *E. coli*, while UG and HMP individuals had higher diversity within the Proteobacteria (Figure 3). In addition, several measures of diversity and richness were significantly altered in DG patients compared to UG patients and HMP controls, as measured by one-way ANOVA. The number of unique OTUs were significantly lower in DG patients compared to UG patients and HMP controls (p = 0.0068). A single *E. coli* OTU comprised a significantly higher percentage of total reads per sample in DG patients compared to UG patients and HMP controls (p = <0.0001). The DG patients also had significantly lower measurements of diversity and richness compared to UG patients and HMP controls, including Chao 1 estimate values (p = 0.0042), Ace richness values (p = 0.0050), and Shannon diversity index values (p = 0.0010). Taken together, these data demonstrate that a subset of NoV-infected patients had an altered intestinal microbiota characterized by a significant loss of diversity and a significant increase in the Proteobacteria *E. coli*.

### Lack of Relationship between Microbiota Disruption and Clinical Variables in Norovirus-infected Patients

We next sought to identify potential associations between clinical patient variables and the differences in microbial community structure encountered between DG and UG patients. The development of mucosal inflammation has been previously associated with increased levels of Proteobacteria [22], [23], [24]. Thus, we measured the level of lactoferrin, a stable marker of inflammation [25], [26] in stool samples from NoV-infected patients. Linear regression analysis failed to show a significant correlation between lactoferrin and Proteobacteria detection (p = 0.3685, Figure S1A), or between lactoferrin and Bacteriodetes detection (p = 0.5971, Figure S1B). Furthermore, lactoferrin levels were not predictive of a disrupted microbiota, and were not significantly different between disrupted and undisrupted patient groups (p = 0.2292).

We next looked at the variables of gender, patient age, and sampling time. Neither gender nor patient age were correlated to elevated Proteobacteria or elevated lactoferrin levels (Figure S2). The level of Proteobacteria (p = 0.3721, Figure S2A) and the level of lactoferrin (p = 0.9050, Figure S2B) were similar in males and females. Increased age was also not significantly correlated to elevated Proteobacteria (p = 0.2711, Figure S3A) or elevated lactoferrin levels (p = 0.9997, Figure S3B). Moreover, sampling time, as defined by the time between illness onset and sample collection, did not influence Proteobacteria (p = 0.1757, Figure S3C) or lactoferrin levels (p = 0.6782, Figure S3D), nor were there any differences in sampling time by patient age (p = 0.7916) or gender (p = 0.1957). The highly altered microbiota of DG patients could not be explained by differences in patient age (p = 0.3245) or sample collection time (p = 0.0690). Furthermore, PCOA showed no apparent clustering by gender, age, lactoferrin level, or sampling time (Figure S4). The relative abundance of specific bacterial phyla and families were compared using two-tailed t-tests, to determine if elevated inflammation was associated with specific members of the community. Patients were separated into two groups, of “high” and “low” lactoferrin, based on a cutoff of 40 µg/g of lactoferrin in stool. No differences were found in the levels of the phyla Bacteriodetes, Firmicutes, and Proteobacteria, or in the families *Enterobacteriaceae*, *Pseudomonadaceae*, *Pasteurellaceae*, *Alcaligenaceae*, *Neisseriaceae*, and *Desulfovibrionaceae* between the high and low lactoferrin groups. Specifically, no differences were detected when comparing all 38 NoV patients or when comparing only within the UG patients. Therefore, it does not appear specific bacterial families could be responsible for the elevated inflammation detected in some UG patients.

Additionally, some alternate patient groupings were used to further examine patient variables. Lactoferrin levels in patients with greater than 10% Proteobacteria were not different than those with less than 10% Proteobacteria (p = 0.3400) as measured by a two-tailed t-test. Furthermore patients were then placed into three putative groups based on Proteobacteria content, <10%, 10–30%, and >30% to compare patient variables using ANOVA. No significant differences were found in lactoferrin levels (p = 0.4835), sampling time (p = 0.2860), or patient age (p = 0.1026) between these groups. Additionally, NoV patients from several epidemiologic clusters were examined, but no correlation was found amongst samples from a given cluster and disruption of the microbiota. In summary, gender, age, lactoferrin levels, and sampling time did not explain the differences seen between UG and DG patients.

### Diversity and Virulence Potential of *E. coli* from Norovirus Patients

To determine if the increase in *E. coli* seen in the DG patients represented outgrowth of bacteria with specific characteristics, *E. coli* was isolated from a subset of patients. The diversity of *E. coli* within and between representative patients of the DG and UG groups was determined using ribotype analysis, and the *E. coli* were assayed for the presence of virulence factors normally seen in enteropathogenic strains. Ribotype diversity was examined within 9 patients (5 undisrupted, 4 disrupted) by using 42–47 cultured *E. coli* isolates per patient. All patients examined harbored a dominant clone of *E. coli*, comprising about 80–100% of all isolates per patient (Table 2). Most ribotypes were found exclusively in either disrupted or undisrupted patient groups. However, several ribotypes were shared between patients, including one shared amongst several individuals, which was the dominant type in 4 of 5 undisrupted patients. This ribotype was not found in any disrupted patients. Additionally, one ribotype was found that was unique to disrupted patients, and one that was detected in both disrupted and undisrupted patients. To investigate pathogenic potential in *E. coli* isolates, a Luminex virulence gene assay designed to detect several pathogenic types of *E. coli* was used [27]. We tested 22 unique *E. coli* isolates cultured from 4 disrupted patients, plus several known pathogenic strains to serve as controls. All 22 samples were negative for each of the 9 virulence genes in this assay (data not shown). In summary, *E. coli* ribotypes were clonal and largely unique to patient groups, but none encoded known pathogenic virulence genes.

**Table 2:** Summary of PCR-ribotypes detected in *Escherichia coli* cultured from several Norovirus patients.

Group	Patient ID	Num. ofIsolates	Ribotype	Num. ofEach Type	Percentageof Each Type
UG	C02S2	44	1	40	90.9%
			2	2	4.5%
			3	1	2.3%
			4	1	2.3%
UG	C06S4	44	1	36	81.8%
			5	6	13.6%
			6	2	4.5%
UG	C07S1	47	7	42	89.4%
			8	1	2.1%
			9	1	2.1%
			10	1	2.1%
			1	1	2.1%
			11	1	2.1%
UG	C15S2	46	1	46	100.0%
UG	C31S2	42	1	34	81.0%
			12	5	11.9%
			13	1	2.4%
			14	1	2.4%
			15	1	2.4%
DG	C06S3	44	16	43	97.7%
			17	1	2.3%
DG	C08S1	44	16	44	100.0%
DG	C31S1	44	18	44	100.0%
DG	C39S3	44	7	36	81.8%
			19	7	15.9%
			20	1	2.3%

Abbreviations: UG, Norovirus patient with an undisrupted microbiota; DG, Norovirus patient with a disrupted microbiota.

Individuals with Norovirus infection contained a dominant type of *E. coli* (80–100% of isolates examined were a single type). Additionally, some PCR ribotypes were shared amongst multiple Norovirus patients, but most types were unique to each patient and each group. Ribotypes were assigned based upon allele combinations seen in Table S5.

## Discussion

This study has established that highly altered gut communities can be detected in a subset of patients with NoV gastroenteritis. Approximately 1 of 5 patients had significant microbiota alterations, resulting in a loss of diversity and increased Proteobacteria. Disruption of the microbiota is a feature of multiple diseases, seen previously following gastrointestinal infection by pathogenic bacteria and parasites [27]–[29]. This study demonstrates that disruption can also be seen following Norovirus gastroenteritis. A diverse gut microbiota is important for maintaining protection from pathogenic infection, by means of colonization resistance [28]. Gut microbial diversity may offer protection from viral diarrhea [29], [30], and loss of diversity may help promote viral infections.

Elevated Proteobacteria is a common feature in patients with an altered microbiota, and could be viewed as a marker for disruption. The current study was the first examination of the diversity and pathogenic potential of elevated Proteobacteria in NoV-infected patients. Intestinal inflammation can be associated with increased Proteobacteria in Crohn’s disease [31] and ulcerative colitis [32], but elevated Proteobacteria can also occur without inflammation. We investigated lactoferrin levels as a marker for inflammation to determine if disruption in NoV patients was linked to inflammation, but did not see correlations between lactoferrin and Proteobacteria. Lupp *et al* found that elevated inflammation during infection can be associated with microbiota disruption, particularly elevated *E. coli* levels [33]. While the current study also found highly elevated *E. coli* levels, no link was found to intestinal inflammation, indicating that multiple mechanisms may contribute to elevated intestinal Proteobacteria levels. Norovirus is not considered an inflammatory pathogen, which may explain the lack of association. Illness severity measurements were unavailable for the patients in the current study. Therefore it was not possible to investigate potential links between inflammation and severity, such as diarrhea or vomiting frequency. Of the several variables that were available, including patient age, gender, sample collection time, and viral load, these variables did not correlate with increases in Proteobacteria and microbiota disruption. *E. coli* was the major component of the elevated Proteobacteria in NoV-infected patients, which has been seen previously at high levels in disrupted communities [34]. Also similar to previous studies, we found the *E. coli* in NoV-infected patients to be clonal [35]. In addition, *E. coli* ribotypes were largely unique to either disrupted or undisrupted patients. Infection by pathogenic *E. coli* variants has been known to disrupt the microbiota [36]. However, the isolates from disrupted NoV patients showed no pathogenic potential and likely represented an outgrowth of commensal organisms. The lack of common virulence markers suggests elevated *E. coli* does not arise from any overt pathogenic ability in these strains.

Microbiota disruption may introduce long lasting effects on health; however the impact of disruption on health is not clearly understood. Disruption and elevated Proteobacteria [6], [37], [38], have been linked to health problems, including IBS, a serious, non-inflammatory medical condition characterized by abnormal bowel habits, abdominal pain, constipation, and diarrhea [39]. A prior episode of acute gastrointestinal infection can cause a specific form of IBS called post-infectious IBS (PI-IBS). Bacterial gastroenteritis has previously been linked to PI-IBS following pathogenic *E. coli* [20], *Salmonella*, and *Campylobacter* [40] infections. Fewer studies have investigated the link between viral gastroenteritis and PI-IBS, but two recent reports specifically found an increased incidence of PI-IBS following NoV gastroenteritis [17], [18]. The current study has demonstrated that microbiota disruption can exist in patients following NoV infection; however additional studies are needed to address if microbiota disruption can increase the risk of long-term illness following episodes of viral gastroenteritis. The samples examined in the current study had limited patient medical data available, and did not specify details such as the use of antibiotics prior to illness, or measurements of the duration of illness. Only a single sample per patient was available. Additional samples from before infection and at various times following illness onset were unavailable, so it was not possible to determine the baseline microbiota prior to illness or the path of recovery. Future studies to address these limitations could include the use of human volunteers to monitor the gut microbiota before and after NoV infection to determine specific responses to infection. Longitudinal follow-up could then establish the risk of developing long-term health problems. It is possible that specific types of microbiota disruption could predict an elevated risk of developing complications, and identify patients that may benefit from probiotic therapies available to treat microbiota disruption [41], [42], [43]. In conclusion, this work provides a foundation for further studies to improve understanding of the interactions of NoV infection and the gut microbiota, and how these interactions can influence human health.

## Materials and Methods

### Sample Collection

Stool samples were collected by the Virginia Division of Consolidated Laboratory Services (DCLS) between February and April 2010 from adult individuals (Table S1) with confirmed NoV infection. A single sample for each patient was collected. Samples were de-identified, then grouped into potential clusters or outbreaks based on close geographic proximity at the time of illness onset. The dates of both illness onset and sample collection were recorded and the length of time between onset and collection was used to calculate days post-onset (DPO), as a measurement of sampling time. Patient gender and age were also collected. A diagnostic RT-PCR [44] performed at DCLS was used to identify NoV-positive samples, and initial genogroup classification. This study was approved by the Human Subjects Committee at the University of Michigan. The requirement for informed consent for the use of these samples was waived by the Human Subjects Committee at the University of Michigan.

### Human NoV Genotyping and Genome Quantification

Further classification by NoV genotype and determination of genome levels was performed as follows. Total RNA was extracted from 10% stool suspensions (0.05–0.1 g stool in 0.5–1 ml of phosphate-buffered saline) using QIAamp™ Viral RNA Mini Kit (Qiagen) according the manufacturer’s recommendations and eluted in 60 µl AVE-buffer (Qiagen). For genotyping, the ORF-1/2 junction or region D ORF-2 cDNA was amplified by conventional OneStep RT-PCR (Qiagen) using described conditions [45], [46]. Genogroup II-specific cDNA was amplified using primers CapC and CapD1 [46], or primers NV107a and NV117 [45]. Genogroup I-specific cDNA was amplified using primers NV65a or NV120 [45]. All cDNAs were sequenced and genogroups were confirmed by blast search and genotypes using the automated NoV typing tool [47].

For the quantification of Genogroup II NoV, a OneStep Real Time - PCR (qRT-PCR) assay was performed on an Applied Biosystems 7500 Fast Real-Time PCR System using previously reported primers, TaqMan® probe, and qRT-PCR conditions [45]. Briefly, a 25 µl reaction mixture was prepared from 10 µl of the extracted total RNA, 4.5 mM MgCl_2_, 10 mM of each dNTP, 0.6 µM of both the sense and the antisense primer (NV107a and NV117), 0.2 µM of the TaqMan probe TM3, 0.1 µM, 1×ROX Reference Dye (Invitrogen), and 1 µl of OneStep RT-PCR Enzyme Mix (QIAGEN). The TM3 probe was dual-labeled with 5′- and 3′- reporter dye FAM and the 3′-quencher dye TAMRA (Eurofins MWG Operon). HuNoV-RNA was reverse transcribed (30 min at 50°C) followed by heat inactivation and activation of the HotStar polymerase by incubation at 95°C for 15 min. Amplification was carried out for 45 cycles with 15 sec at 94°C and 1 min at 56°C. To quantify the genome equivalents, an external standard curve was established using a 10-fold serial dilution ranging from 4e8 to 4e2 copies of a plasmid containing Genogroup II-cDNA (AY032605, nt 5007–5100).

### Controls

Sequences from healthy control subjects were obtained from the NIH Human Microbiome Project (HMP) and the 16S Short Read Archive (SRA) [48]. All sequences were from the V3–V5 hypervariable region of the 16S rRNA gene and generated from barcoded pyrosequencing experiments conducted at the Baylor College of Medicine. Subjects eligible for the HMP had no signs of gastrointestinal disease, were not on antibiotics, and were otherwise healthy. Full details of the criteria for inclusion in the HMP is described in the HMP Core Microbiome Sampling Protocol HMP-07-001, which can be found at: http://www.hmpdacc.org/doc/HMP_Clinical_Protocol.pdf.

Specific sequences used are listed in Table S3. All samples were from study phs000228, from the Human Microbiome Project 16S rRNA 454 Clinical Production Phase I (SRP002395), and utilized HMP Core Microbiome Sampling Protocol A (HMP-A). Sequence data can be obtained here: http://www.ncbi.nlm.nih.gov/projects/gap/cgi-bin/study.cgi?study_id=phs000228.v3.p1.

HMP patient age data was obtained through a controlled access data request from the database of Genotypes and Phenotypes (dbGaP) [49] at the National Center for Biotechnology Information.

### DNA Extraction

DNA extraction was performed using the Roche MagNA Pure Compact system. Briefly, stool samples were suspended in a mixture of sterile PBS (pH 7.4, Invitrogen, Carlsbad, CA) and Roche Bacterial Lysis Buffer (Roche Diagnostics GmbH, Mannheim, Germany). Cells were disrupted by bead-beating for 1 minute, followed by treatment with 50 µL of proteinase K for 10 minutes at 65°C. Samples were then bead beat for an additional 1 minute, then heat inactivated for 10 minutes at 95°C. Further processing of the samples on the MagNA pure compact was performed according to the protocol included with the Roche MagNA Pure Nucleic Acid Isolation Kit I.

DNA was then quantified using a NanoDrop 1000 spectrophotometer (NanoDrop, Wilmington, DE) and stored at −20°C.

### Pyrosequencing

Stool derived DNA samples were submitted for 16S rRNA gene amplification and pyrosequencing in two separate batches at the Human Genome Sequencing Center at Baylor University College of Medicine in Houston, TX and the University of Michigan DNA Sequencing Core in Ann Arbor, MI.

Amplification of the V3–V5 region of the 16S rRNA gene was accomplished using the Broad HMP protocol (HMP MDG Default Protocol v4.2), which can be found at: http://www.hmpdacc.org/doc/HMP_MDG_454_16S_Protocol_V4_2_102109.pdf.

Amplified PCR products were checked for quality on a 2% agarose gel for visual verification, and then each sample was individually quantified using the Quant-It PicoGreen dsDNA kit (Molecular Probes, Eugene, OR). Each sample was diluted to normalize concentrations before pooling. The pooled sample was then checked on a Bioanalyzer 2100 machine (Agilent, Santa Clara, CA), using a DNA1000 lab chip (Agilent, Santa Clara, CA) to verify sample purity prior to amplification by emulsion PCR and pyrosequencing.

Sequences are available via MG-RAST (ID numbers 4501002.3–4501035.3) at: http://metagenomics.anl.gov/linkin.cgi?project=1957.

### Pyrosequencing Data Processing and Analysis

Analysis of 454 Pyrosequencing data was performed using mothur (version 1.20) [50]. Specifically, the standard operating procedure instructions on the mothur website were followed for pyrosequencing data processing. The processing SOP can be found here: http://www.mothur.org/wiki/Schloss_SOP.

mothur was used for determining operational taxonomic units, for comparing community structure, and for classification of 16S rRNA sequences. Classifications were determined by aligning sequences to the Ribosomal Database Project (Michigan State University, East Lansing, MI). After trimming to remove low quality base calls, sequences were filtered based on size, and all reads less than 196 nucleotides were removed. Reads with one or more ambiguous calls were also removed. Only sequences containing the reverse primer (V5) were used in the analysis.

Sequences were assigned to OTUs based on 97% sequence identity prior to classification by the Ribosomal Database Project classifier. Classified OTUs were then used to determine the relative abundance of bacterial phyla in each sample and for statistical comparisons between samples.

Principle coordinates analysis (PCOA) was used to assess community similarity among all samples. More specifically, PCOA was used to analyze a Yue and Clayton-based distance matrix representing the relative abundance of OTUs in each community. These distances were displayed visually in 2-dimensional space.

Statistical analyses were performed using GraphPad Prism version 5 (GraphPad Software, San Diego California USA). These included linear regression analysis, box and whiskers plots, one-way ANOVA, and unpaired, two-tailed T-tests.

### Inflammation Assay

Lactoferrin was used as a marker for inflammation in stool [25]. An ELISA-based quantitative kit, IBD-SCAN (TECHLAB, Blacksburg, VA), was used per the manufacturer’s specifications.

### Cultivation and Isolation of *E. coli* from Stool

Selective culturing of *E. coli* from NoV patient stool samples was performed by plating onto EC-MUG Media, a combination of Difco EC Medium (Becton, Dickinson, and Company, Sparks, MD) and MUG (4-Methylumbelliferyl-β-D-glucuronide). EC Medium is selective for *E. coli*, and MUG is a compound that, when hydrolyzed by the *E. coli*-specific enzyme ß-D-glucoronidase, produces a flurogen, 4-methylumbelliferone, that glows blue under ultraviolet light [51].

Sterile PBS was used to suspend small amounts of stool for serial dilutions at 10^−1^, 10^−2^, 10^−3^, and 10^−4^ using 75 µL of suspension spread on each plate before growth overnight at 37°C. The following day, individual colonies with blue fluorescence under ultraviolet light were selected using sterile toothpicks. Selected individual colonies were used for colony-pick PCR during the PCR Ribotyping assay.

### PCR Ribotyping of Cultivated *E. coli*

PCR Ribotyping was used to type cultured *E. coli* isolated from stool of NoV patients. This technique used PCR primers complementary to conserved regions of the 16S and 23S genes in the ribosomal operon, and amplified the intergenic region between those genes [52]. This intergenic region can vary in size, and because *E. coli* contains seven different copies of this operon, a pattern of amplicon sizes can be detected from a single isolate and used to identify specific types. Amplicons were grouped into alleles based upon size (Table S4), which allowed for ribotypes to be assigned to unique combinations of alleles (Table S5).

Primers used for ribotyping were: Forward 5′ – CAGGGCATCCACCGTGT –3′, Reverse 5′ – GTGAAGTCGTAACAAGG –3′, and were adapted from a previously published pair designed to work with *Salmonella* [53]. The forward primer also included a 6-FAM label on 5′ end to allow amplicons to be used with fragment analysis genotyping. Genotyping was performed at the Genome Sequencing Center at the University of Michigan.

PCR was performed using Illustra PuReTaq Ready-To-Go PCR beads (GE Healthcare, Little Chalfont, Buckinghamshire, UK) with the following cocktail per reaction: 4 µL of each forward and reverse labeled primer (at 2 pmol/µL), and 15 uL sterile water. This cocktail was added to the PCR beads and mixed gently prior to cycling. Single colony picks of cultured *E. coli* were suspended gently in the cocktail and used as DNA templates for the PCR reaction. Cycling conditions for were the same as published previously [53].

Ribotyping controls used included three *E. coli* isolates, with the unique amplicon sizes, in basepairs, listed in parentheses. 042 (443, 535), K-12 MG1665 (443, 520, 526, 529, 535), and CFT-073 (443, 444, 520, 521, 526, 529, 538). Expected amplicon sizes for controls were determined from sequences available in Genbank.

PCR products were diluted 1∶100 in sterile water then 1 µL of diluted sample was added to a plate containing formamide and a ROX1000 size standard, and then submitted for fragment analysis typing. Size standard estimations were rounded off to whole nucleotides for analysis and publication.

### Virulence Gene Assay for Cultivated *E. coli*

*E. coli* isolates were selected for a virulence gene assay on a Luminex panel [27]. The virulence genes included markers for Avian Pathogenic *E. coli* (APEC): *aatA*; Enteraggregative *E. coli* (EAEC): *aaiC*; Enterohemorrhagic *E. coli* (EHEC): *stx1* and *stx2*; Enteropathogenic *E. coli* (EPEC): *eae* and *bfpA*; Enterotoxigenic *E. coli* (ETEC): *elt*, *est*; and Enteroinvasive *E. coli* (EIEC): *ipaH*. Strains of pathogenic *E. coli* were used as controls, including EHEC (Sakai and TW14359), ETEC (H10407), EPEC (E2348/69), Urinary Pathogenic *E. coli* (CFT073), and the non-pathogenic isolate K-12. All cultured isolates and control strains were randomized and de-identified prior to testing.

## Supporting Information

Figure S1 **Relationship between lactoferrin levels and phylum-level detection.** Linear regression analysis found no significant relationship between gut inflammation, as measured by lactoferrin levels, and phylum-level disruptions in the microbiota, including with Proteobacteria (A) and Bacteriodetes (B).
(TIF)

Figure S2 **Relationship between gender and Proteobacteria detection.** Gender was not significantly correlated to increased Proteobacteria detection (A), or lactoferrin level (B) as determined by unpaired two-tailed T-tests.
(TIF)

Figure S3 **Relationship of patient age and sample collection time to Proteobacteria detection.** Linear regressions confirm that donor age was not correlated to increases in Proteobacteria (A) or the level of lactoferrin detected (B). Also, sampling time, as measured by days post onset of illness symptoms, was not significantly correlated to differences in the level of Proteobacteria detected (C), or with inflammation level, as measured by lactoferrin (D).
(TIF)

Figure S4 **Principle coordinates analysis reveals no apparent cluster by patient variables.** No apparent clustering can be seen when comparing OTU diversity and richness between patients using gender (A), age (B), lactoferrin (inflammation) level (C), or sampling time as measured by days post onset (D). Each symbol represents the community structure for one individual. Age is separated by patients younger or older than the median age of 80. Inflammation is separated by patients higher or lower than 40 ng/g(mL) of lactoferrin in stool. Sampling time is divided by patients higher or lower than the median sampling time of 1 day post onset for stool sample collection. HMP controls are not included in the lactoferrin and sampling time plots because data for those variables was unavailable or not applicable. PCOA plots were generated using the Yue and Clayton based distance matrix with OTUs defined by 97% or greater similarity.
(TIF)

Table S1 **Summary of clinical data associated with individual Norovirus-infected patients.**
(DOCX)

Table S2 **Sequencing quality and alpha diversity measurements for all NoV and HMP patients in this study.**
(DOCX)

Table S3 **Summary of data for healthy control patients from the Human Microbiome Project (HMP).**
(DOCX)

Table S4 **Size bins used for sorting PCR-ribotyping generated amplicons.**
(DOCX)

Table S5 **Allele combinations used for PCR ribotype assignments.** Each cultured *Escherichia coli* isolate was assigned a ribotypes based on a unique allele combination. Alleles letter codes and amplicon sizes used are listed in Table S4.
(DOCX)
