# Supplementary figures and images for: Disruption of the Human Gut Microbiota following Norovirus Infection
Source: PLoS One. 2012 Oct 30;7(10):e48224. doi: 10.1371/journal.pone.0048224 (PMC3484122; doi:10.1371/journal.pone.0048224)

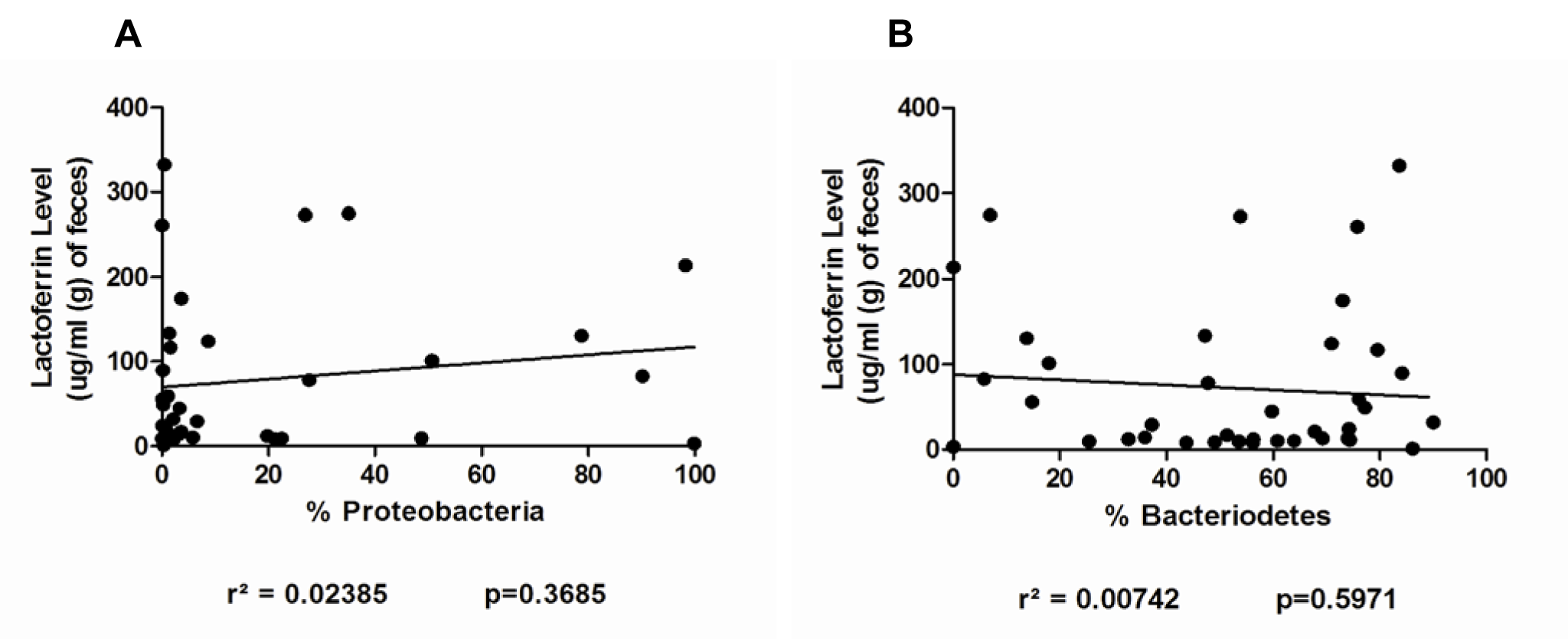

Supplement: Figure S1 — Relationship between lactoferrin levels and phylum-level detection. Linear regression analysis found no significant relationship between gut inflammation, as measured by lactoferrin levels, and phylum-level disruptions in the microbiota, including with Proteobacteria (A) and Bacteriodetes (B). (TIF) [file pone.0048224.s001.tif]

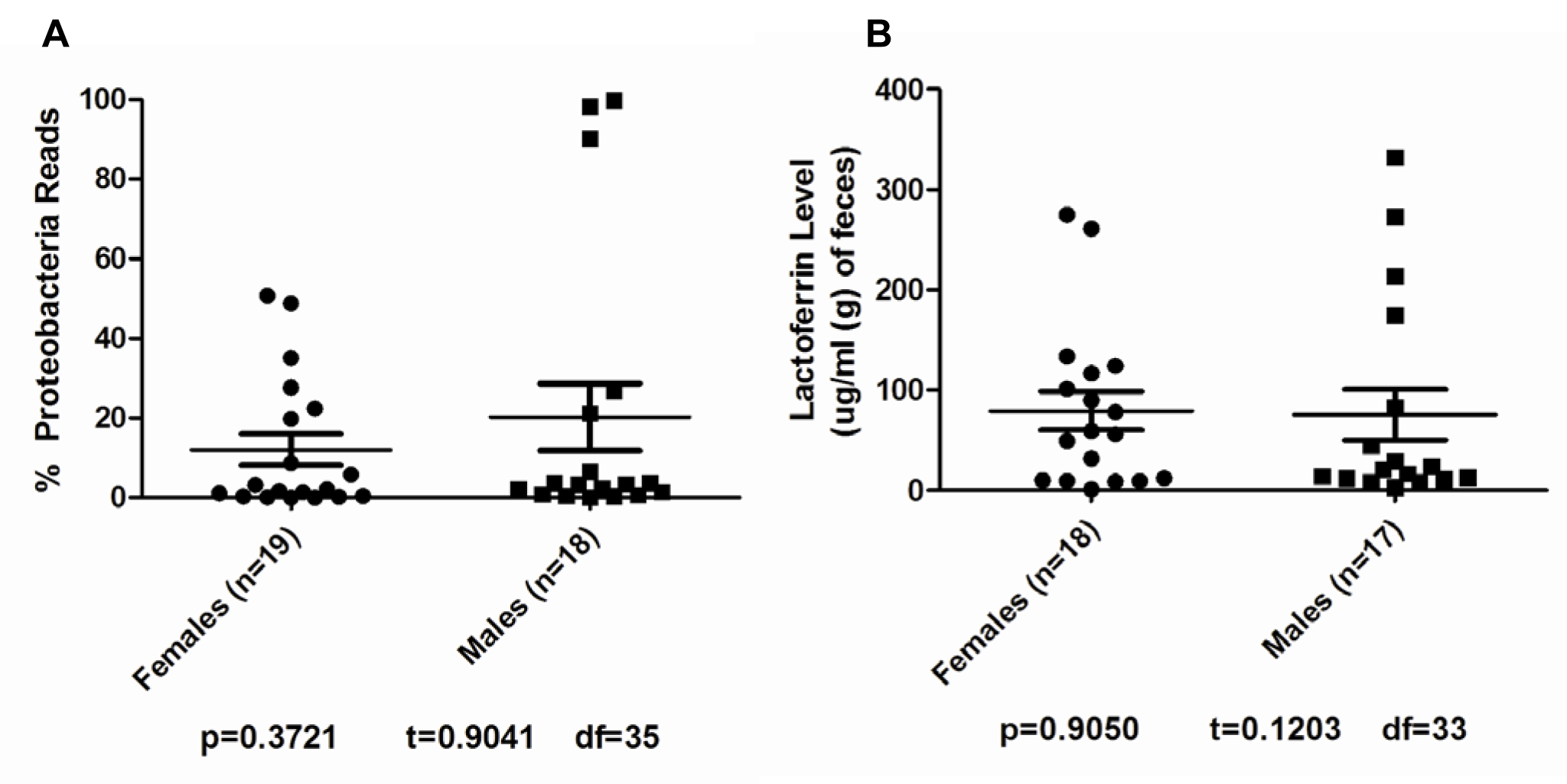

Supplement: Figure S2 — Relationship between gender and Proteobacteria detection. Gender was not significantly correlated to increased Proteobacteria detection (A), or lactoferrin level (B) as determined by unpaired two-tailed T-tests. (TIF) [file pone.0048224.s002.tif]

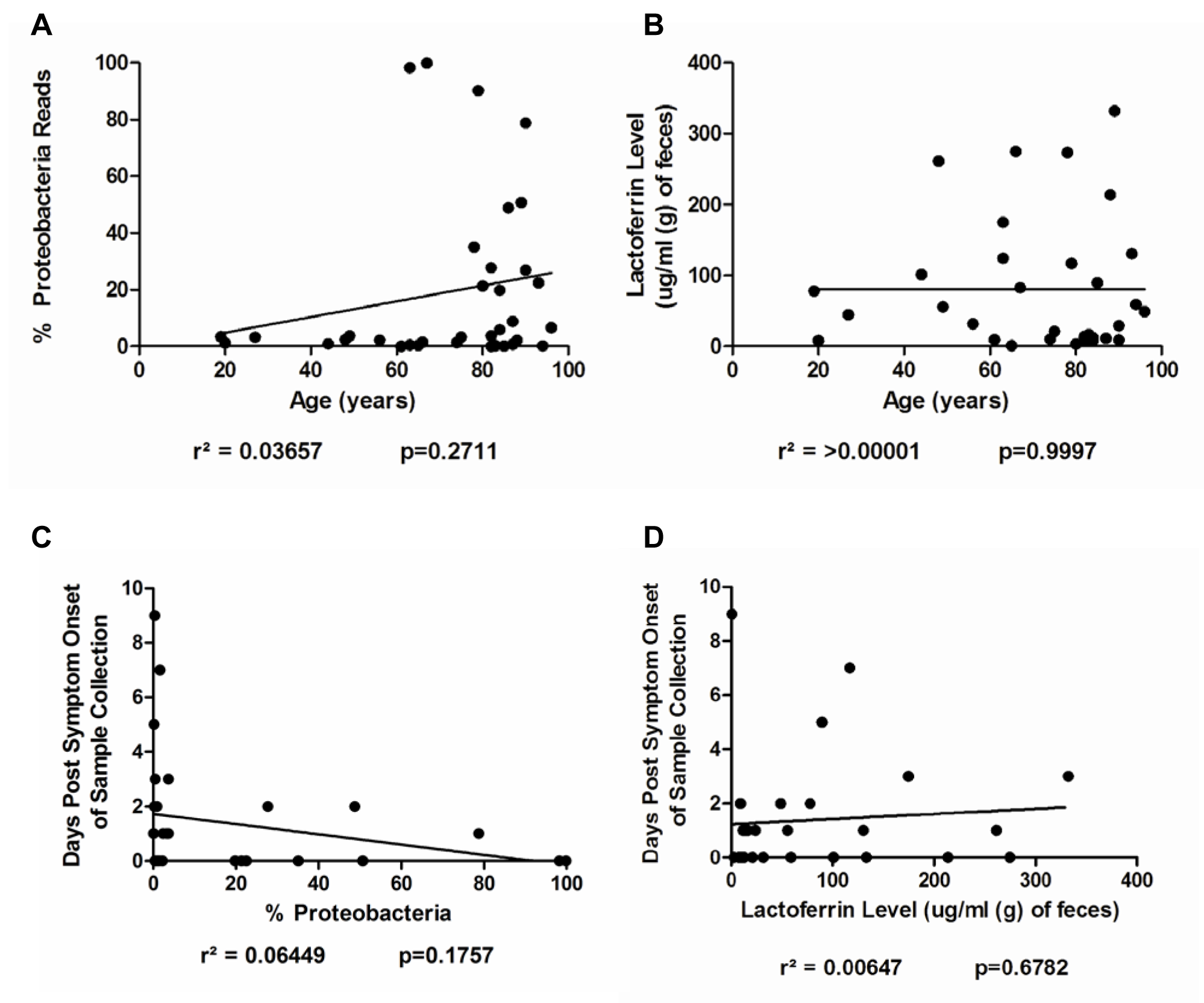

Supplement: Figure S3 — Relationship of patient age and sample collection time to Proteobacteria detection. Linear regressions confirm that donor age was not correlated to increases in Proteobacteria (A) or the level of lactoferrin detected (B). Also, sampling time, as measured by days post onset of illness symptoms, was not significantly correlated to differences in the level of Proteobacteria detected (C), or with inflammation level, as measured by lactoferrin (D). (TIF) [file pone.0048224.s003.tif]

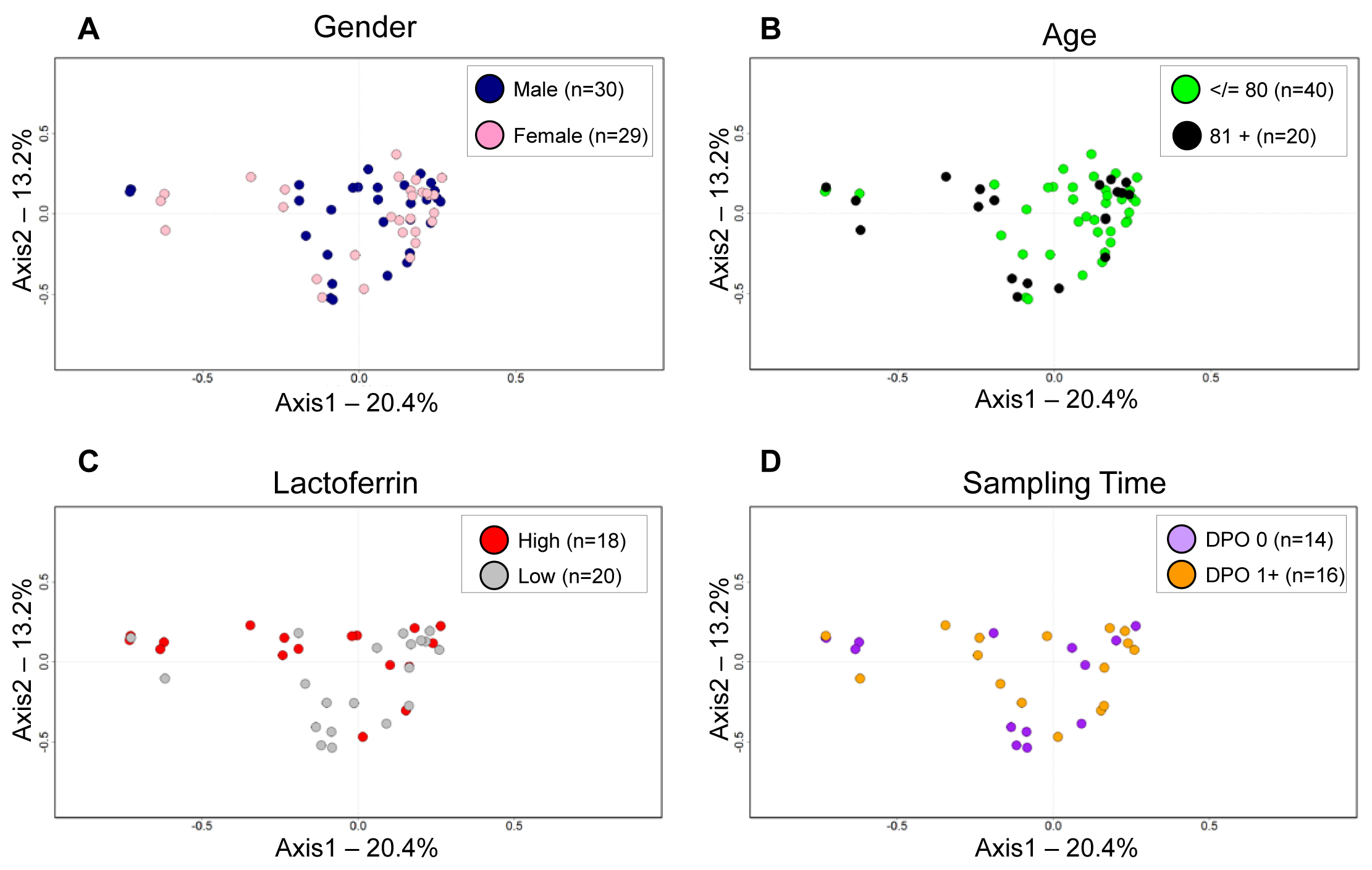

Supplement: Figure S4 — Principle coordinates analysis reveals no apparent cluster by patient variables. No apparent clustering can be seen when comparing OTU diversity and richness between patients using gender (A), age (B), lactoferrin (inflammation) level (C), or sampling time as measured by days post onset (D). Each symbol represents the community structure for one individual. Age is separated by patients younger or older than the median age of 80. Inflammation is separated by patients higher or lower than 40 ng/g(mL) of lactoferrin in stool. Sampling time is divided by patients higher or lower than the median sampling time of 1 day post onset for stool sample collection. HMP controls are not included in the lactoferrin and sampling time plots because data for those variables was unavailable or not applicable. PCOA plots were generated using the Yue and Clayton based distance matrix with OTUs defined by 97% or greater similarity. (TIF) [file pone.0048224.s004.tif]
