# Supplementary material for: Disruption of the Human Gut Microbiota following Norovirus Infection
Source: PLoS One. 2012 Oct 30;7(10):e48224. doi: 10.1371/journal.pone.0048224 (PMC3484122; doi:10.1371/journal.pone.0048224)
Supplement: Table S1 — Summary of clinical data associated with individual Norovirus-infected patients. (DOCX) [file pone.0048224.s005.docx]

|  |  |  |  |  | **Lactoferrin** | **Percentage** | **Norovirus** | **Viral** |
| --- | --- | --- | --- | --- | --- | --- | --- | --- |
| **ID** | **Group** | **Age** | **Gender** | **DPO** | **ng/(g)mL** | **Proteobacteria** | **Genotype** | **Load** |
| C06S3 | DG | 90 | ND | 1 | 130.5 | 78.76 | GII.4 + GI | 3.61E+08 |
| C08S1 | DG | 78 | F | 0 | 274.8 | 35.07 | GII.4 | ND |
| C10S1 | DG | 79 | M | ND | 82.8 | 90.18 | GII.4 | 1.01E+05 |
| C12S1 | DG | 67 | M | 0 | 3.3 | 99.88 | GII.4 | 6.88E+04 |
| C31S1 | DG | 63 | M | 0 | 213.7 | 98.25 | GII.4 | 5.25E+04 |
| C38S1 | DG | 89 | F | 0 | 101.1 | 50.67 | GII.4 + GI | 1.63E+06 |
| C39S3 | DG | 86 | F | 2 | 9.6 | 48.76 | GII.4 | 3.36E+07 |
| C01S1 | UG | ND | F | 1 | 261.3 | 0.05 | GII.4 | 4.66E+06 |
| C02S2 | UG | 48 | M | 1 | 12.1 | 2.4 | GII.4 | 6.97E+08 |
| C03S1 | UG | 84 | F | 0 | 12.1 | 19.81 | GII.4 | ND |
| C03S4 | UG | 82 | M | 1 | 16.7 | 3.72 | GII.4 | 1.78E+05 |
| C05S1 | UG | 83 | F | 2 | 49.2 | 0.25 | GII.4 | 1.65E+06 |
| C06S2 | UG | 96 | M | ND | 29.3 | 6.63 | GII.4 | 1.45E+05 |
| C06S4 | UG | 93 | F | 0 | 9.3 | 22.45 | GII.4 | 3.83E+05 |
| C07S1 | UG | 90 | M | ND | 273.1 | 26.9 | GII.4 | ND |
| C08S3 | UG | 66 | M | 0 | 13.1 | 1.42 | GII.4 | 6.32E+06 |
| C09S1 | UG | ND | F | 7 | 116.9 | 1.65 | GII.4 | 5.35E+04 |
| C12S2 | UG | 80 | M | 0 | 8.3 | 21.28 | GII.4 | ND |
| C15S2 | UG | 84 | F | ND | 10 | 5.88 | GII.4 | 6.90E+05 |
| C17S1 | UG | 74 | F | 0 | 133.5 | 1.41 | GII.4 | 1.71E+06 |
| C18S1 | UG | ND | F | 9 | 1.1 | 0.36 | GII.4 | 2.15E+05 |
| C21S1 | UG | 65 | M | 0 | 11.2 | 0.41 | GII.4 | 7.44E+05 |
| C21S2 | UG | 87 | M | 0 | 21.3 | 0.67 | GII.4 | 1.24E+05 |
| C21S3 | UG | 75 | M | 1 | 14 | 3.26 | GII.4 | 4.20E+06 |
| C23S1 | UG | 82 | F | 2 | 78.1 | 27.72 | GII.4 + GI | 1.03E+04 |
| C26S2 | UG | 19 | M | ND | 44.8 | 3.34 | GII.4 | 1.00E+05 |
| C26S3 | UG | 27 | F | ND | ND | 3.19 | GII.4 | ND |
| C30S2 | UG | 87 | F | ND | 124.3 | 8.76 | GII.4 | 2.54E+07 |
| C31S2 | UG | 88 | F | 0 | 31.7 | 2.14 | GII.4 | 7.57E+05 |
| C31S3 | UG | 56 | M | 0 | 8.2 | 2.24 | GII.4 | 5.10E+04 |
| C31S4 | UG | 20 | F | 0 | 59.3 | 1.14 | GII.4 | 5.18E+04 |
| C32S1 | UG | 94 | F | 5 | 89.9 | 0.22 | GII.4 | 1.62E+07 |
| C34S1 | UG | 49 | M | 3 | 174.7 | 3.72 | GII.4 | 8.54E+04 |
| C34S2 | UG | 63 | M | 3 | 332.2 | 0.45 | GII.4 | 7.55E+04 |
| C39S2 | UG | 44 | M | 2 | ND | 0.91 | GII.4 | ND |
| C40S1 | UG | 61 | F | ND | 8.9 | 0.02 | GII.4 | 7.35E+04 |
| C42S1 | UG | 82 | M | 1 | 24.1 | 0.08 | GII.4 | 1.32E+08 |
| C33S2 | Outlier | 85 | F | 1 | 55.9 | 0.15 | GII.4 | ND |

Abbreviations: DG, Norovirus patient with a disrupted microbiota; UG, Norovirus patient with an undisrupted microbiota; ND, not determined; DPO, days post onset (time between illness onset and sample collection); M, male; F, female.

The percentage Proteobacteria column indicates the percentage of reads in that sample classified on the phylum level as *Proteobacteria* from the total reads per sample.

Viral load shows the number of NoV genome copies per mL.

A single outlier (sample C33S2) was excluded from both the DG and UG sample sets.
