# Supplementary material for: Disruption of the Human Gut Microbiota following Norovirus Infection
Source: PLoS One. 2012 Oct 30;7(10):e48224. doi: 10.1371/journal.pone.0048224 (PMC3484122; doi:10.1371/journal.pone.0048224)
Supplement: Table S2 — Sequencing quality and alpha diversity measurements for all NoV and HMP patients in this study. (DOCX) [file pone.0048224.s006.docx]

| \| **Sample** \|  \|  \| **Total** \| **Freq** \|  \| **Estimated OTU Richness^d^** \| \|  \|  \| \| --- \| --- \| --- \| --- \| --- \| --- \| --- \| --- \| --- \| --- \| \| **ID** \| **Group** \| **Read^a^** \| **OTUs^b^** \| **OTU2^c^** \| **ESC** \| **Chao 1** \| **Ace** \| **Inv Simpson** \| **Shannon^e^** \| \| C06S3 \| DG \| 7237 \| 148 \| 5625 \| 99.0% \| 246 (201, 330) \| 388 (326, 472) \| 1.64 \| 1.26 \| \| C08S1 \| DG \| 6961 \| 115 \| 2321 \| 99.4% \| 165 (138, 220) \| 216 (181, 269) \| 4.12 \| 2.08 \| \| C10S1 \| DG \| 6578 \| 78 \| 5880 \| 99.4% \| 174 (119, 302) \| 207 (162, 275) \| 1.25 \| 0.62 \| \| C12S1 \| DG \| 4209 \| 7 \| 4202 \| 99.9% \| 12 (8, 39) \| 22 (8, 170) \| 1.00 \| 0.02 \| \| C31S1 \| DG \| 1203 \| 7 \| 1178 \| 99.6% \| 17 (9, 59) \| 0 (0,0) \| 1.04 \| 0.12 \| \| C38S1 \| DG \| 5857 \| 133 \| 2765 \| 98.7% \| 306 (223, 467) \| 495 (405, 614) \| 3.85 \| 2.09 \| \| C39S3 \| DG \| 6132 \| 132 \| 2906 \| 99.0% \| 230 (182, 327) \| 358 (299, 438) \| 3.53 \| 2.07 \| \| C01S1 \| UG \| 8592 \| 354 \| 0 \| 97.5% \| 769 (637, 961) \| 1653 (1455, 1885) \| 7.00 \| 2.84 \| \| C02S2 \| UG \| 6699 \| 265 \| 3 \| 98.1% \| 457 (384, 574) \| 644 (563, 746) \| 8.63 \| 3.07 \| \| C03S1 \| UG \| 1752 \| 64 \| 56 \| 97.8% \| 249 (132, 568) \| 583 (426, 808) \| 9.70 \| 2.71 \| \| C03S4 \| UG \| 8120 \| 251 \| 138 \| 98.6% \| 397 (341, 488) \| 556 (487, 646) \| 7.30 \| 2.93 \| \| C05S1 \| UG \| 4362 \| 177 \| 0 \| 97.6% \| 346 (227, 465) \| 721 (605, 869) \| 5.68 \| 2.49 \| \| C06S2 \| UG \| 8141 \| 313 \| 297 \| 97.9% \| 610 (508, 764) \| 888 (775, 1029) \| 11.83 \| 3.29 \| \| C06S4 \| UG \| 8426 \| 194 \| 563 \| 98.9% \| 402 (310, 568) \| 500 (429, 593) \| 3.68 \| 2.24 \| \| C07S1 \| UG \| 3450 \| 168 \| 865 \| 97.3% \| 343 (267, 476) \| 541 (452, 658) \| 7.27 \| 2.70 \| \| C08S3 \| UG \| 8995 \| 227 \| 33 \| 98.9% \| 349 (299, 433) \| 455 (398, 529) \| 5.84 \| 2.78 \| \| C09S1 \| UG \| 5163 \| 190 \| 24 \| 97.9% \| 443 (334, 633) \| 651 (553, 776) \| 1.97 \| 1.70 \| \| C12S2 \| UG \| 11227 \| 553 \| 2268 \| 97.0% \| 1296 (1097, 1569) \| 2416 (2181, 2686) \| 12.04 \| 3.47 \| \| C15S2 \| UG \| 6903 \| 266 \| 169 \| 98.0% \| 540 (436, 708) \| 814 (713, 938) \| 6.58 \| 2.87 \| \| C17S1 \| UG \| 3474 \| 191 \| 17 \| 96.5% \| 489 (365, 698) \| 913 (766, 1098) \| 12.48 \| 3.06 \| \| C18S1 \| UG \| 3578 \| 109 \| 0 \| 98.3% \| 227 (167, 348) \| 360 (292, 454) \| 3.24 \| 1.80 \| \| C21S1 \| UG \| 3666 \| 73 \| 0 \| 99.0% \| 163 (109, 299) \| 199 (157, 264) \| 1.82 \| 1.26 \| \| C21S2 \| UG \| 3274 \| 166 \| 19 \| 97.2% \| 337 (263, 468) \| 508 (426, 616) \| 4.41 \| 2.31 \| \| C21S3 \| UG \| 2818 \| 160 \| 18 \| 96.8% \| 338 (259, 481) \| 695 (582, 837) \| 8.79 \| 3.00 \| \| C23S1 \| UG \| 5686 \| 330 \| 1507 \| 96.6% \| 765 (618, 989) \| 1225 (1084, 1394) \| 8.44 \| 3.03 \| \| C26S2 \| UG \| 9457 \| 354 \| 81 \| 98.1% \| 605 (522, 729) \| 874 (773, 999) \| 17.87 \| 3.56 \| \| C26S3 \| UG \| 7141 \| 257 \| 2 \| 98.2% \| 479 (395, 613) \| 706 (613, 824) \| 13.69 \| 3.28 \| \| C30S2 \| UG \| 4556 \| 281 \| 0 \| 96.3% \| 660 (524, 873) \| 1174 (1020, 1360) \| 11.61 \| 3.28 \| \| C31S2 \| UG \| 5885 \| 62 \| 105 \| 99.6% \| 90 (72, 137) \| 86 (72, 120) \| 2.52 \| 1.43 \| \| C31S3 \| UG \| 7289 \| 257 \| 1 \| 98.4% \| 521 (414, 702) \| 737 (644, 851) \| 16.39 \| 3.54 \| \| C31S4 \| UG \| 4118 \| 142 \| 0 \| 98.6% \| 217 (180, 290) \| 297 (252, 360) \| 5.34 \| 2.57 \| \| C32S1 \| UG \| 3243 \| 90 \| 0 \| 98.6% \| 166 (125, 257) \| 232 (186, 301) \| 4.11 \| 2.08 \| \| C34S1 \| UG \| 9054 \| 357 \| 0 \| 97.9% \| 723, 599, 910) \| 975 (866, 1106) \| 13.59 \| 3.47 \| \| C34S2 \| UG \| 11354 \| 196 \| 0 \| 99.2% \| 361 (290, 485) \| 470 (405, 556) \| 5.00 \| 2.38 \| \| C39S2 \| UG \| 7730 \| 254 \| 14 \| 98.3% \| 602 (463, 833) \| 798 (696, 925) \| 6.35 \| 2.75 \| \| C40S1 \| UG \| 4348 \| 313 \| 0 \| 95.7% \| 726 (585, 940) \| 1379 (1212, 1578) \| 7.65 \| 3.08 \| \| C42S1 \| UG \| 4780 \| 150 \| 0 \| 98.5% \| 265 (211, 368) \| 358 (303, 432) \| 8.45 \| 2.70 \| \| C33S2 \| Outlier \| 4770 \| 362 \| 4 \| 96.0% \| 695 (586, 858) \| 994 (882, 1129) \| 20.74 \| 3.85 \| \| HMP-1 \| HMP \| 6868 \| 361 \| 2 \| 96.9% \| 855 (694, 1094) \| 1382 (1219, 1576) \| 11.12 \| 3.24 \| \| HMP-2 \| HMP \| 7451 \| 191 \| 0 \| 98.7% \| 344 (280, 454) \| 516 (440, 614) \| 6.01 \| 2.36 \| \| HMP-3 \| HMP \| 6571 \| 389 \| 0 \| 96.6% \| 823 (688, 1021) \| 1369 (1215, 1553) \| 19.93 \| 3.69 \| \| HMP-4 \| HMP \| 5214 \| 106 \| 0 \| 98.8% \| 274 (188, 452) \| 508 (408, 642) \| 4.08 \| 1.85 \| \| HMP-5 \| HMP \| 7699 \| 145 \| 0 \| 98.9% \| 315 (238, 455) \| 615 (507, 755) \| 5.26 \| 2.13 \| \| HMP-6 \| HMP \| 5138 \| 341 \| 1 \| 96.5% \| 699 (576, 886) \| 1145 (1015, 1299) \| 15.30 \| 3.76 \| \| HMP-7 \| HMP \| 6094 \| 268 \| 0 \| 97.5% \| 555 (450, 720) \| 926 (806, 1073) \| 11.33 \| 3.17 \| \| HMP-8 \| HMP \| 8696 \| 220 \| 0 \| 98.6% \| 520 (397, 728) \| 882 (761, 1029) \| 9.42 \| 2.78 \| \| HMP-9 \| HMP \| 6326 \| 162 \| 0 \| 98.6% \| 314 (247, 436) \| 395 (334, 478) \| 1.54 \| 1.14 \| \| HMP-10 \| HMP \| 5322 \| 134 \| 2 \| 98.7% \| 281 (208, 426) \| 385 (317, 476) \| 4.04 \| 2.12 \| \| HMP-11 \| HMP \| 6606 \| 222 \| 0 \| 98.2% \| 460 (366, 616) \| 627 (578, 791) \| 7.67 \| 2.73 \| \| HMP-12 \| HMP \| 5929 \| 282 \| 12 \| 97.5% \| 547 (450, 700) \| 874 (764, 1010) \| 9.69 \| 3.27 \| \| HMP-13 \| HMP \| 6322 \| 53 \| 3 \| 99.6% \| 78 (62, 123) \| 114 (89, 157) \| 2.86 \| 1.39 \| \| HMP-14 \| HMP \| 3492 \| 54 \| 1 \| 99.2% \| 117 (77, 227) \| 172 (129, 240) \| 4.85 \| 1.87 \| \| HMP-15 \| HMP \| 4425 \| 76 \| 0 \| 99.3% \| 107 (89, 151) \| 113 (93, 154) \| 4.66 \| 1.97 \| \| HMP-16 \| HMP \| 3976 \| 187 \| 0 \| 97.5% \| 363 (288, 493) \| 654 (557, 777) \| 9.80 \| 3.03 \| \| HMP-17 \| HMP \| 4863 \| 126 \| 0 \| 98.8% \| 207 (167, 286) \| 294 (245, 363) \| 4.93 \| 2.24 \| \| HMP-18 \| HMP \| 4361 \| 131 \| 0 \| 98.6% \| 245 (188, 360) \| 326 (271, 403) \| 4.07 \| 2.21 \| \| HMP-19 \| HMP \| 11145 \| 242 \| 0 \| 98.9% \| 488 (389, 653) \| 704 (611, 820) \| 11.32 \| 3.05 \| \| HMP-20 \| HMP \| 9202 \| 281 \| 0 \| 98.5% \| 572 (461, 751) \| 669 (585, 778) \| 7.52 \| 3.02 \| \| HMP-21 \| HMP \| 9883 \| 323 \| 0 \| 98.5% \| 544 (466, 663) \| 710 (631, 810) \| 10.35 \| 3.21 \| \| HMP-22 \| HMP \| 17631 \| 323 \| 9 \| 99.0% \| 651 (540, 820) \| 1170 (1035, 1330) \| 4.49 \| 2.30 \| |
| --- | --- | --- | --- | --- | --- | --- | --- | --- | --- | --- | --- | --- | --- | --- | --- | --- | --- | --- | --- | --- | --- | --- | --- | --- | --- | --- | --- | --- | --- | --- | --- | --- | --- | --- | --- | --- | --- | --- | --- | --- | --- | --- | --- | --- | --- | --- | --- | --- | --- | --- | --- | --- | --- | --- | --- | --- | --- | --- | --- | --- | --- | --- | --- | --- | --- | --- | --- | --- | --- | --- | --- | --- | --- | --- | --- | --- | --- | --- | --- | --- | --- | --- | --- | --- | --- | --- | --- | --- | --- | --- | --- | --- | --- | --- | --- | --- | --- | --- | --- | --- | --- | --- | --- | --- | --- | --- | --- | --- | --- | --- | --- | --- | --- | --- | --- | --- | --- | --- | --- | --- | --- | --- | --- | --- | --- | --- | --- | --- | --- | --- | --- | --- | --- | --- | --- | --- | --- | --- | --- | --- | --- | --- | --- | --- | --- | --- | --- | --- | --- | --- | --- | --- | --- | --- | --- | --- | --- | --- | --- | --- | --- | --- | --- | --- | --- | --- | --- | --- | --- | --- | --- | --- | --- | --- | --- | --- | --- | --- | --- | --- | --- | --- | --- | --- | --- | --- | --- | --- | --- | --- | --- | --- | --- | --- | --- | --- | --- | --- | --- | --- | --- | --- | --- | --- | --- | --- | --- | --- | --- | --- | --- | --- | --- | --- | --- | --- | --- | --- | --- | --- | --- | --- | --- | --- | --- | --- | --- | --- | --- | --- | --- | --- | --- | --- | --- | --- | --- | --- | --- | --- | --- | --- | --- | --- | --- | --- | --- | --- | --- | --- | --- | --- | --- | --- | --- | --- | --- | --- | --- | --- | --- | --- | --- | --- | --- | --- | --- | --- | --- | --- | --- | --- | --- | --- | --- | --- | --- | --- | --- | --- | --- | --- | --- | --- | --- | --- | --- | --- | --- | --- | --- | --- | --- | --- | --- | --- | --- | --- | --- | --- | --- | --- | --- | --- | --- | --- | --- | --- | --- | --- | --- | --- | --- | --- | --- | --- | --- | --- | --- | --- | --- | --- | --- | --- | --- | --- | --- | --- | --- | --- | --- | --- | --- | --- | --- | --- | --- | --- | --- | --- | --- | --- | --- | --- | --- | --- | --- | --- | --- | --- | --- | --- | --- | --- | --- | --- | --- | --- | --- | --- | --- | --- | --- | --- | --- | --- | --- | --- | --- | --- | --- | --- | --- | --- | --- | --- | --- | --- | --- | --- | --- | --- | --- | --- | --- | --- | --- | --- | --- | --- | --- | --- | --- | --- | --- | --- | --- | --- | --- | --- | --- | --- | --- | --- | --- | --- | --- | --- | --- | --- | --- | --- | --- | --- | --- | --- | --- | --- | --- | --- | --- | --- | --- | --- | --- | --- | --- | --- | --- | --- | --- | --- | --- | --- | --- | --- | --- | --- | --- | --- | --- | --- | --- | --- | --- | --- | --- | --- | --- | --- | --- | --- | --- | --- | --- | --- | --- | --- | --- | --- | --- | --- | --- | --- | --- | --- | --- | --- | --- | --- | --- | --- | --- | --- | --- | --- | --- | --- | --- | --- | --- | --- | --- | --- | --- | --- | --- | --- | --- | --- | --- | --- | --- | --- | --- | --- | --- | --- | --- | --- | --- | --- | --- | --- | --- | --- | --- | --- | --- | --- | --- | --- | --- | --- | --- | --- | --- | --- | --- | --- | --- | --- | --- | --- | --- | --- | --- | --- | --- | --- | --- | --- | --- | --- | --- | --- | --- | --- | --- | --- | --- | --- | --- | --- | --- | --- | --- | --- | --- | --- | --- | --- | --- | --- | --- | --- | --- | --- | --- | --- | --- | --- | --- | --- | --- | --- | --- | --- | --- | --- | --- | --- | --- | --- | --- | --- | --- | --- | --- | --- | --- | --- | --- | --- | --- | --- | --- | --- | --- | --- | --- | --- | --- | --- | --- | --- | --- | --- | --- | --- | --- | --- | --- | --- | --- | --- | --- | --- | --- | --- | --- | --- | --- | --- | --- | --- | --- | --- | --- | --- |

Abbreviations: DG, disrupted group - Norovirus patient with a disrupted microbiota; UG, undisrupted group – Norovirus patient with an undisrupted microbiota; HMP, healthy control patient; OTU, operational taxonomic unit; ESC, estimated sample coverage.

^a^Total number of sequence reads per sample.

^b^Total number of unique OTUs detected (as calculated by mothur at a 3% distance level).

^c^Number of times OTU2 was detected per sample.

^d^Values in brackets for OTU richness estimates represent 95% confidence intervals, as calculated by mothur.

^e^Shannon diversity values are calculated by mothur (at 3% distance).

Sample C33S2 was treated as an outlier and was not included as part of the disrupted or undisrupted patient groups.
