# Supplementary material for: Disruption of the Human Gut Microbiota following Norovirus Infection
Source: PLoS One. 2012 Oct 30;7(10):e48224. doi: 10.1371/journal.pone.0048224 (PMC3484122; doi:10.1371/journal.pone.0048224)
Supplement: Table S3 — Summary of data for healthy control patients from the Human Microbiome Project (HMP). (DOCX) [file pone.0048224.s007.docx]

|  | **NCBI** |  |  | **Percentage** |  | **Accession** | **Anonymized** |
| --- | --- | --- | --- | --- | --- | --- | --- |
| **ID** | **ID^a^** | **Age** | **Gender** | **Proteobacteria** | **SRA^b^** | **Run ID^c^** | **Name^d^** |
| HMP1 | 33409 | 22 | F | 1.60 | SRS012191 | SRR048044 | 158013734 |
| HMP2 | 44205 | 29 | F | 4.16 | SRS022987 | SRR048059 | 158114885 |
| HMP3 | 43128 | 24 | M | 0.20 | SRS021910 | SRR048072 | 158155345 |
| HMP4 | 42759 | 29 | M | 0.10 | SRS021541 | SRR048083 | 158216035 |
| HMP5 | 44142 | 37 | F | 0.04 | SRS022924 | SRR048095 | 158236265 |
| HMP6 | 44265 | 24 | F | 6.95 | SRS023047 | SRR048110 | 158276726 |
| HMP7 | 41337 | 31 | M | 0.39 | SRS020119 | SRR048128 | 158398106 |
| HMP8 | 41394 | 32 | M | 1.44 | SRS020176 | SRR048143 | 158418336 |
| HMP9 | 44640 | 24 | M | 0.43 | SRS023422 | SRR048157 | 158438567 |
| HMP10 | 45729 | 25 | M | 4.31 | SRS024511 | SRR048158 | 159389382 |
| HMP11 | 43698 | 30 | F | 1.99 | SRS022480 | SRR048171 | 158458797 |
| HMP12 | 43632 | 27 | F | 0.79 | SRS022414 | SRR048189 | 158742018 |
| HMP13 | 45069 | 29 | F | 0.40 | SRS023851 | SRR048207 | 158883629 |
| HMP14 | 44706 | 25 | M | 2.40 | SRS023488 | SRR048223 | 158964549 |
| HMP15 | 68092 | 28 | M | 2.16 | SRS048083 | SRR048234 | 159146620 |
| HMP16 | 77131 | 23 | M | 9.32 | SRS057122 | SRR048247 | 159166850 |
| HMP17 | 34761 | 22 | F | 0.28 | SRS013543 | SRR048260 | 159227541 |
| HMP18 | 34980 | 25 | M | 2.18 | SRS013762 | SRR048289 | 159389382 |
| HMP19 | 62299 | 34 | F | 4.33 | SRS042290 | SRR048434 | 159085930 |
| HMP20 | 76665 | 33 | M | 0.06 | SRS056656 | SRR048448 | 159288231 |
| HMP21 | 74497 | 27 | M | 0.08 | SRS054488 | SRR048464 | 159632143 |
| HMP22 | 76514 | 23 | F | 0.79 | SRS056505 | SRR048477 | 159753524 |

^a^NCBI biosample ID number.

^b^Short read archive ID number at dbGaP.

^c^Run ID for individual sample in short read archive.

^d^dbGaP patient donor ID.

Sequence information for HMP samples can be located using the NCBI ID at: http://www.ncbi.nlm.nih.gov/biosample/.

The percentage Proteobacteria column indicates the percentage of reads in that sample classified on the phylum level as *Proteobacteria* from the total reads per sample.
