# Supplementary material for: Disruption of the Human Gut Microbiota following Norovirus Infection
Source: PLoS One. 2012 Oct 30;7(10):e48224. doi: 10.1371/journal.pone.0048224 (PMC3484122; doi:10.1371/journal.pone.0048224)
Supplement: Table S4 — Size bins used for sorting PCR-ribotyping generated amplicons. (DOCX) [file pone.0048224.s008.docx]

|  | **Allele** | **Ribotype Size Bins** | |  |
| --- | --- | --- | --- | --- |
|  | **Code** | **Lower** | **Upper** | **Note** |
| 1 | A | 113 | 115 | 1 |
| 2 | B | 139 | 141 | 1 |
| 3 | C | 157 | 159 | 1 |
| 4 | D | 213 | 216 | 1 |
| 5 | E | 281 | 283 | 1 |
| 6 | F | 285 | 287 | 1 |
| 7 | G | 293 | 295 | 1 |
| 8 | H | 422 | 424 | 2 |
| 9 | I | 425 | 427 | 1 |
| 10 | J | 430 | 432 | 1,2 |
| 11 | K | 432 | 434 | 1,2 |
| 12 | L | 435 | 437 | 1,2 |
| 13 | M | 438 | 439 | 1,2 |
| 14 | N | 441 | 444 | 1,2 |
| 15 | O | 446 | 449 | 1 |
| 16 | P | 481 | 483 | 1,2 |
| 17 | Q | 498 | 500 | 2 |
| 18 | R | 508 | 510 | 2 |
| 19 | S | 512 | 514 | 1,2 |
| 20 | T | 515 | 518 | 1,2 |
| 21 | U | 519 | 522 | 2 |
| 22 | V | 522 | 524 | 2 |
| 23 | W | 525 | 527 | 1,2 |
| 24 | X | 529 | 531 | 1,2 |
| 25 | Y | 534 | 537 | 1,2 |
| 26 | Z | 537 | 539 | 2 |
| 27 | AA | 540 | 542 | 2 |
| 28 | AB | 542 | 544 | 1 |
| 29 | AC | 552 | 556 | 1 |
| 30 | AD | 560 | 562 | 1 |
| 31 | AE | 567 | 569 | 1 |
| 32 | AF | 570 | 572 | 1 |
| 33 | AG | 583 | 585 | 1 |
| 34 | AH | 586 | 588 | 1 |
| 35 | AI | 592 | 594 | 1 |
| 36 | AJ | 612 | 614 | 1 |
| 37 | AK | 622 | 624 | 1 |
| 38 | AL | 636 | 638 | 1 |
| 39 | AM | 660 | 662 | 1 |
| 40 | AN | 697 | 699 | 1 |
| 41 | AO | 701 | 703 | 1 |

1 – Detected in cultured isolates.

2 – Detected in GenBank genome sequences.
