# Supplementary material for: Disruption of the Human Gut Microbiota following Norovirus Infection
Source: PLoS One. 2012 Oct 30;7(10):e48224. doi: 10.1371/journal.pone.0048224 (PMC3484122; doi:10.1371/journal.pone.0048224)
Supplement: Table S5 — Allele combinations used for PCR ribotype assignments. Each cultured Escherichia coli isolate was assigned a ribotypes based on a unique allele combination. Alleles letter codes and amplicon sizes used are listed in Table S4. (DOCX) [file pone.0048224.s009.docx]

| **Ribotype** | **Allele Code** |
| --- | --- |
| 1 | N, Y |
| 2 | F, J, T |
| 3 | F, J, T, AH |
| 4 | N, O, AB |
| 5 | N, W, X, Y |
| 6 | L, N, Y |
| 7 | N, X, Y |
| 8 | F, J |
| 9 | G, I, M, N |
| 10 | N |
| 11 | N, X, Y, AD, AF |
| 12 | N, AE, AF, AI, AK |
| 13 | A, B, AC |
| 14 | C, P, AI, AJ, AM |
| 15 | AC, AJ, AL |
| 16 | A, B, D, AC, AN, AO |
| 17 | B, AC, AN, AO |
| 18 | AC, AJ, AL, AM |
| 19 | E, I, M, S, AG |
| 20 | E, I, M, N, S, Y |
